# Upregulation of the proto-oncogene Bmi-1 predicts a poor prognosis in pediatric acute lymphoblastic leukemia

**DOI:** 10.1186/s12885-017-3049-3

**Published:** 2017-01-25

**Authors:** Hong-Xia Peng, Xiao-Dan Liu, Zi-Yan Luo, Xiao-Hong Zhang, Xue-Qun Luo, Xiao Chen, Hua Jiang, Ling Xu

**Affiliations:** 1 Department of Hematology, Guangzhou Women and Children’s Medical Center, Guangzhou Medical University, 9 Jinsui Road, Guangzhou, Guangdong 510623 China; 2 Division of Birth Cohort Study, Guangzhou Women and Children’s Medical Center, Guangzhou Medical University, Guangzhou, China; 3 Department of Pediatrics, The First Affiliated Hospital of Sun Yat-sen University, Guangzhou, China; 4 Department of Pediatrics, Zhuzhou Central Hospital, Zhuzhou, China

**Keywords:** Bmi-1, Pediatric acute lymphoblastic leukemia, Sall4, Prognosis

## Abstract

**Background:**

Bmi-1, the B cell-specific moloney murine leukemia virus insertion site 1, is a member of the Polycomb-group (PcG) family and acts as an oncogene in various tumors; however, its expression related to the prognosis of pediatric patients with acute lymphoblastic leukemia (ALL) has not been well studied.

**Methods:**

The Bmi-1 expression levels in the bone marrow of 104 pediatric ALL patients and 18 normal control subjects were determined by using qRT-PCR. The association between the Bmi-1 expression and the clinicopathological characteristics of pediatric ALL patients was analyzed, and the correlation between Bmi-1 and the prognosis of pediatric ALL was calculated according to the Kaplan–Meier method. Furthermore, the association between Bmi-1 expression and its transcriptional regulator Sall4 was investigated.

**Results:**

Compared to normal control subjects, patients with primary pediatric ALL exhibited upregulated levels of Bmi-1. However, these levels were sharply decreased in patients who achieved complete remission. A significant positive association between elevated Bmi-1 levels and a poor response to prednisone as well as an increased clinical risk was observed. Patients who overexpressed Bmi-1 at the time of diagnosis had a lower relapse-free survival (RFS) rate (75.8%), whereas patients with lower Bmi-1 expression had an RFS of 94.1%. Furthermore, in ALL patients, the mRNA expression of Bmi-1 was positively correlated to the mRNA expression of Sall4a.

**Conclusions:**

Taken together, these data suggest that Bmi-1 could serve as a novel prognostic biomarker in pediatric primary ALL and may be partially regulated by Sall4a. Our study also showed that Bmi-1 could serve as a new therapeutic target for the treatment of pediatric ALL.

**Electronic supplementary material:**

The online version of this article (doi:10.1186/s12885-017-3049-3) contains supplementary material, which is available to authorized users.

## Background

Acute lymphoblastic leukemia (ALL) is a common pediatric malignant tumor characterized by the overproduction and accumulation of immature lymphoid cells and accounts for nearly 25% of all cancers among children younger than 15 years old [1]. Although treatment options for ALL have significantly expanded in the last 10 years, 15–20% of ALL patients cannot achieve long-term remission, and relapse remains a challenge in treating pediatric ALL. Therefore, identifying novel prognostic markers is an urgent issue in ALL [2, 3].

The Bmi-1 (B cell-specific moloney murine leukemia virus integration site 1) gene is a recognized oncogene of the Polycomb-group (PcG) family and was originally identified via retroviral insertional mutagenesis in *Eμ*-c-myc transgenic mice that were infected with the Moloney murine leukemia virus [4, 5]. The human Bmi-1 gene is located at chr.10p13, which has been shown to undergo rearrangements in malignant T cell lymphomas and chromosomal translocation in infant leukemia [6–8]. Bmi-1 has been implicated to play a critical role in a number of biological pathways, including stem cell self-renewal [9–11], DNA damage response [12, 13], cell cycle [14] and senescence [15, 16].

Recently, Bmi-1 has been the focus of significant clinical interest because studies have demonstrated its upregulation in various malignancies such as non-small cell lung cancer [17], breast cancer [18, 19] and colorectal cancer [20], as well as hematological malignancies including mantle cell lymphoma [21], B cell non-Hodgkin’s lymphoma [22] and acute myeloid leukemia (AML) [23]. Abnormal overexpression of Bmi-1 has also been proposed to be involved in tumor invasion, metastasis, cancer therapy failure, and poor prognosis. For example, elevated Bmi-1 levels were observed in 38.7% (29/ 75) cases in nasopharyngeal carcinoma, and its overexpression is correlated to the patients’ survival rate: the 5-year overall survival rate was higher in the Bmi-1-negative group than that in the Bmi-1-positive group (84.2% *vs.*47.6%) [24]. Similar results were also observed in prostate cancer [25, 26], chronic myeloid leukemia [27, 28] and diffuse large B cell lymphomas [29]. Although a relationship between Bmi-1 expression and the prognosis of patients with pediatric ALL has not been determined, the biological functions of Bmi-1 suggest that this protein could play a crucial role in the pathogenesis of pediatric ALL.

In consideration of the important role of Bmi-1 expression in tumorigenesis, the regulation of Bmi-1 is also thought to be essential. Some studies have revealed that Sall4 directly regulates Bmi-1 in both mouse models and human AML cell lines [30, 31]. Consistent with this, a positive correlation between the expression of the Bmi-1 and Sall4 genes was also discovered in the placenta and umbilical cord blood groups [32]. However, to the best of our knowledge, there are no data describing whether Sall4 contributes to the pathogenesis of leukemia.

The current study analyzed the expression and prognostic value of Bmi-1 in pediatric ALL and further elucidated the relationship between Bmi-1 and Sall4. Our results indicated that Bmi-1 was frequently upregulated in patients with ALL compared to healthy subjects, and patients with upregulated Bmi-1 at the time of diagnosis had a lower relapse-free survival (RFS) rate than patients who had lower Bmi-1 expression. In addition, Bmi-1 was observed to be positively correlated to Sall4a. Our data suggest that Bmi-1 could serve as a novel biomarker for the prognostic evaluation of patients with pediatric ALL.

## Methods

### Patients and samples

Tissue samples from 85 ALL patients before initiation of therapy, 19 ALL patients after therapy completion and 18 healthy subjects were collected between July 2006 and June 2009 at the Guangzhou Women and Children’s Medical Center of Guangzhou Medical University and the First Affiliated Hospital of Sun Yat-sen University. The demographics of the patients and healthy donors are summarized in the supplementary data (Additional file 1: Table S1 and Additional file 2: Table S2). Bone marrow was collected from the patients via bone marrow puncture either at the time of diagnosis or during follow-up after treatment. The research protocols were approved by the Ethics Committee of Guangzhou Women and Children’s Medical Center and the First Affiliated Hospital of Sun Yat-sen University. Written informed consent was obtained from the participants’ parents or guardians.

### RNA isolation and quantitative reverse transcription polymerase chain reaction (qRT-PCR)

Total RNA was extracted from patient samples by using TRIzol reagent (Life Technologies, Grand Island, NY) according to the manufacturer’s protocol. The purity and integrity of total RNA were tested to assess the RNA quality. First, the OD ratios at A260/A280 and A260/A230 ranged between 1.8-2; second, the ratio of the 28S and 18S rRNA bands, which were assessed by denaturing gel electrophoresis, was approximately 2:1. For qRT-PCR, cDNA was synthesized from 100 ng total RNA using ABI TaqMan® Reverse Transcription Reagents (Thermo Fisher Scientific Inc., Waltham, MA USA). For first-strand cDNA synthesis, 100 ng of total RNA was used with random hexamer primers, 1× TaqMan RT buffer, 50 U of MultiScribe Reverse Transcriptase and 40 U of RNase inhibitor in a final volume of 20 μl. The mixture was incubated for 10 min at 25 °C, 30 min at 48 °C, and 5 min at 95 °C. Then, qPCR was performed using a Platinum® Quantitative PCR SuperMix-UDG kit (Thermo Fisher Scientific Inc.) according to the standard TaqMan® protocol. The qPCR was performed in a 20 μl PCR reaction containing l μl RT product, 1× PCR SuperMix-UDG, and 100 nM probe. The reactions were performed in a 96-well plate with an initial denaturation at 95 °C for 2 min followed by 40 cycles of 95 °C for 15 s and 60 °C for 30 s. All PCR reactions were run in triplicate with GAPDH used as an internal control. All the primers used are listed in Additional file 3: Table S3. The relative expression of each gene was calculated according to the comparative 2^−ΔΔCt^ method where ΔCt = Ct (target gene) – Ct (GAPDH) and ΔΔCt = ΔCt (sample) − ΔCt (control); the processed data are presented as the fold change of each mRNA.

### Statistical analysis

All results were analyzed using proper statistical methods. Beyond the traditional descriptive statistical analyses, inferential analyses were performed using nonparametric methods. Differences in the mRNA expression between two groups (e.g., control *vs* primary, primary *vs* complete remission (CR), CR *vs* relapse) were analyzed using the Mann–Whitney *U* test for independent unpaired samples and the Wilcoxon test for paired samples. In instances of comparisons among more than two groups (e.g., samples divided into the low-risk group (LR), intermediate risk group (IR) and high-risk group (HR)), the Kruskal-Wallis test was performed first followed by Bonferroni’s correction for multiple comparisons. For categorical variables, the *χ* ^2^ or Fisher exact tests were used, and correlations were determined using the Spearman rank correlation coefficient(r). An analysis of RFS—defined as the time from CR to relapse—was performed according to the Kaplan–Meier method, and comparisons of outcomes among subgroups were performed by using the log-rank test. A two-sided *P* < 0.05 was considered to represent a statistically significant difference. All calculations were performed using GraphPad Prism 6.0 software.

## Results

### Analysis of Bmi-1 expression levels in pediatric ALL patients

**Fig. 1:**
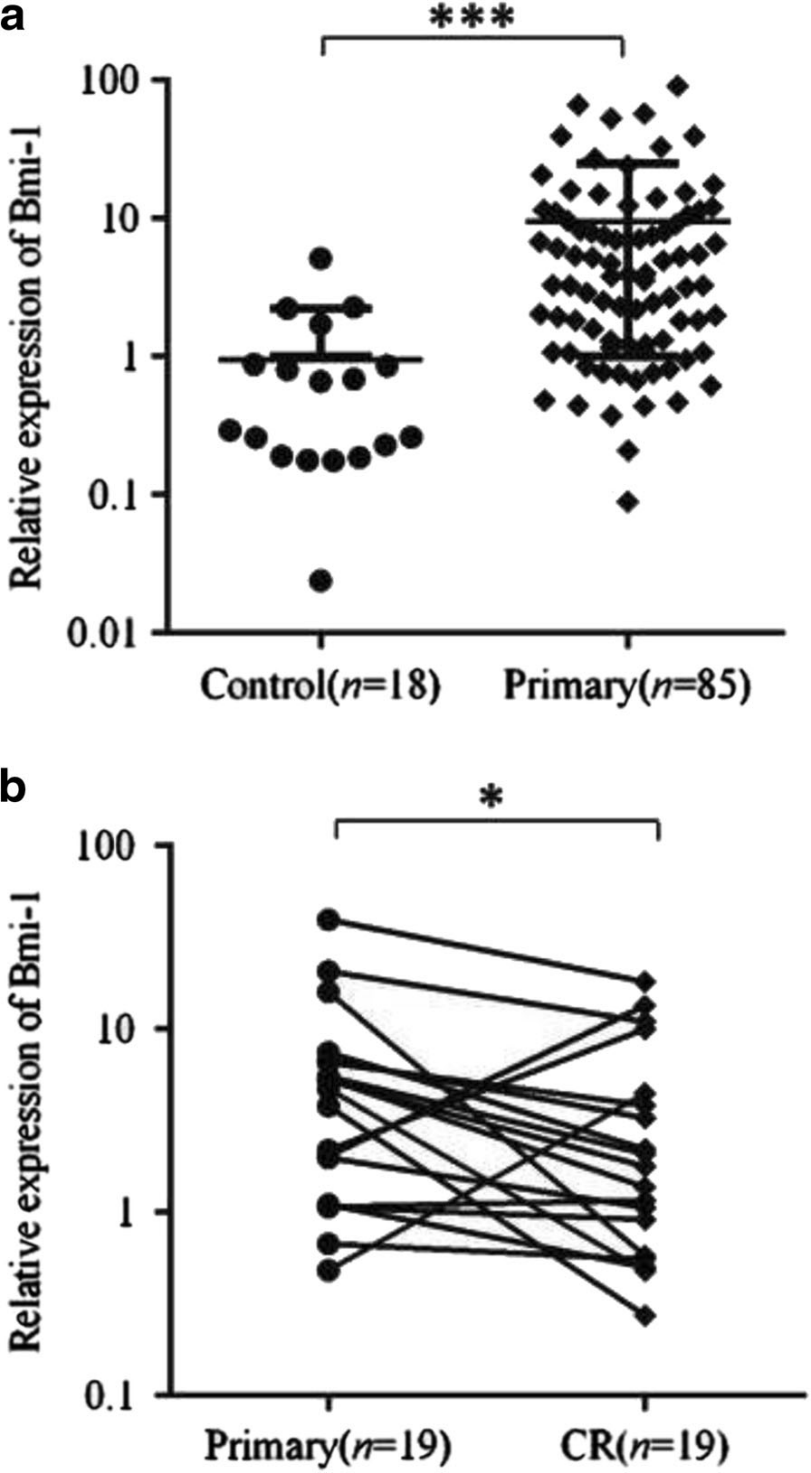
Bmi-1 expression was increased in the pediatric ALL clinical specimens. The qRT-PCR assay was repeated three times and produced similar results for each replicate. The Bmi-1 levels are presented as the means ± standard deviation (M ± SD) and were normalized to the GAPDH levels. **a** The average expression levels of Bmi-1 in pediatric ALL patients (*n* = 85) versus normal control subjects (*n* = 18). **b** The average expression levels of Bmi-1 before and after therapy (*n* = 19) in the paired samples from pediatric ALL patients. ***P < 0.001; *P < 0.05. CR, complete remission

To determine the expression pattern of Bmi-1 in pediatric ALL, 85 bone marrow specimens from pediatric patients with primary ALL and 18 bone marrow specimens from normal subjects were analyzed by using qRT-PCR. Bmi-1 expression was detected in all of the bone marrow samples, with significantly higher expression observed in the primary ALL samples compared with that in the samples from healthy donors (*P* < 0.001, Fig. 1a). Among the 85 ALL samples, 56 (65.9%) cases showed greater than 2-fold upregulation in Bmi-1 expression. To study the changes in Bmi-1 expression before and after therapy treatment, we detected the Bmi-1 levels in pairs of samples from individuals who achieve CR after treatment (*n* = 19). Interestingly, the results found that Bmi-1 expression was sharply decreased in the majority of CR samples (73.7%) after treatment, suggesting that Bmi-1 could be a prognostic indicator (Fig. 1b, *P* = 0.0446). It is noted that the Bmi-1 expression level was still higher than that in normal control subjects although the induction therapy inhibited its expression in these patients to some extent (Additional file 4: Figure S1, *P* = 0.0026). In addition, we assessed several samples by using Western blot. The preliminary data showed that the protein expression levels of Bmi-1 were higher in primary ALL samples than those in the control samples, and Bmi-1 protein expression was slightly decreased in ALL patients who achieved CR, which was consistent with the mRNA expression pattern (data not shown). Therefore, the significant difference in Bmi-1 expression between primary ALL patients and patients who achieved CR implied that Bmi-1 could be used as an important biomarker for clinical prognosis.

### Relationship between Bmi-1 expression and the clinicopathological characteristics of pediatric ALL patients

**Fig. 2:**
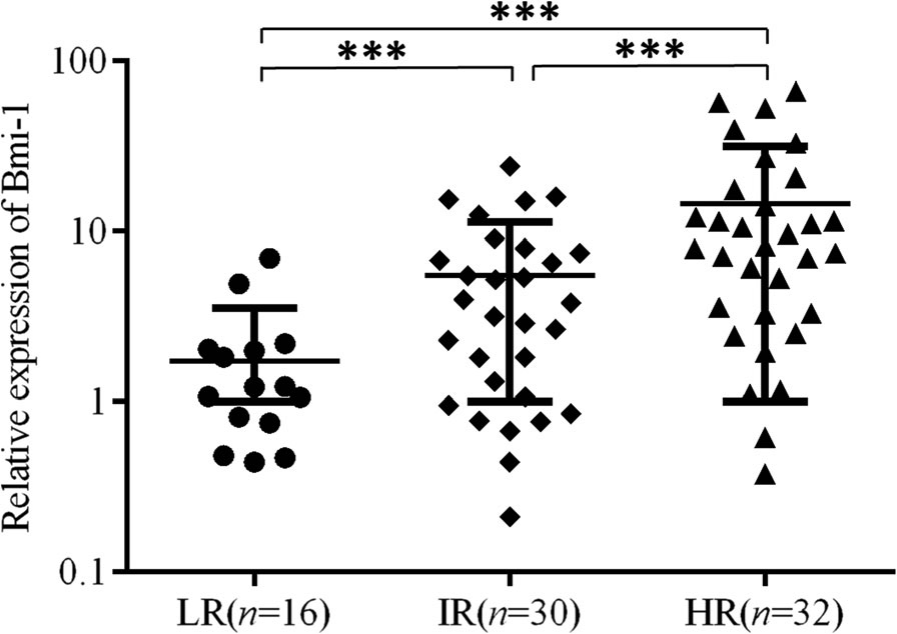
Expression of Bmi-1 mRNA in the low-, intermediate and high-risk groups. ***P < 0.001. LR, low-risk group; IR, intermediate risk group and HR, high-risk group

To determine whether Bmi-1 expression correlates with the clinicopathological characteristics of pediatric ALL patients, we divided the patients into high and low groups based on the median value of Bmi-1 expression among the cohort. Notably, highly expressed Bmi-1 was found to be closely correlated to a poor response to prednisone (*p* = 0.039) and was significantly more prevalent among clinically higher risk groups (*p* = 0.002) (Table 1). To further detect the expression level of Bmi-1 in different clinical risk grade groups, all patients were divided into three hierarchy subgroups (LR, IR, and HR) according to their clinical information (e.g., patient age, initial leukocyte count, chromosomal aberrations, immunophenotype, minimal residual disease and responsiveness to chemotherapy) as described in Yeoh AE et al. [33] and the CCLG-ALL-2008 protocol [34]. The results showed that Bmi-1 expression exhibited an incremental trend in pediatric ALL patients that corresponded to the clinic risk grades: Bmi-1 showed the highest expression in the HR group followed by the IR group and the LR group (Fig. 2, *P* < 0.001). Compared with that in the LR group, the Bmi-1 expression was approximately 3-fold higher in the IR group and almost 8-fold higher in the HR group. However, there were no correlations between Bmi-1 expression and other available pathological data, including gender, age, white blood cell count (WBC), FAB classification and BCR/ABL fusion gene.

**Table 1:** Relationship characteristics of pediatric ALL and Bmi-1 expression level

Characteristics	*n*	Bmi-1		*P* value
		Low expression	High expression	
Age at diagnosis, y				0.100
< 6	43	30 (69.8%)	13 (30.2%)	
≥ 6	42	22 (52.4%)	20 (47.6%)	
Gender				0.161
Male	60	26 (43.3%)	34 (56.7%)	
Female	25	15 (60%)	10 (40%)	
WBC count (×10^9^/L)				0.153
< 50	53	30 (56.6%)	23 (43.4%)	
≥ 50	32	13 (40.6%)	19 (59.4%)	
FAB classification				0.315^a^
L1	34	13 (38.2%)	21 (61.8%)	
L2	47	26 (55.3%)	21 (44.6%)	
L3	4	2 (50%)	2 (50%)	
Immunophenotype				0.281^a^
T	11	4 (36.4%)	7 (63.6%)	
B	66	39 (59%)	27 (41%)	
BCR/ABL				0.329^a^
+	8	3 (37.5%)	5 (62.5%)	
-	64	40 (62.5%)	24 (37.5%)	
Prednisone-test				0.039
PGR	61	36 (59%)	25 (41%)	
PPR	14	4 (28.6%)	10 (71.4%)	
Risk group				0.002^a^
LR	16	14 (87.5%)	2 (12.5%)	
IR	31	16 (51.6%)	15 (48.4%)	
HR	31	10 (32.3%)	21 (67.7%)	

^a^Two-sided Fisher’s exact test

### The influence of Bmi-1 expression on the prognosis of pediatric ALL

Of the 85 initially enrolled patients, 14 patients (16.5%) abandoned treatment after diagnosis, and 4 patients (4.7%) received hematopoietic stem cell transplantation and then ceased contact. The remaining 67 pediatric ALL patients (78.8%) received substantially distinct therapies utilizing the IC-BFM 2002 or VHR-ALL GZCLG protocols according to their clinical risk classification after diagnosis. (The details of treatment regimens were available in Additional file 5: Table S4.) The characteristics of the analyzed pediatric ALL subgroups are listed in Additional file 6: Table S5. Compared with those lost to the follow-up, the analyzed subgroup (treatment group) showed no differences in the distribution of the available parameters (e.g., gender, age, WBC count, FAB classification and BCR/ABL fusion gene). After a median follow-up of 17 months (range from 4 to 42 months), the 3-year RFS was 85.1%. In addition, 10 patients (14.9%) relapsed: 7 patients (10.4%) with isolated BM relapse, 2 patients (3%) with isolated central nervous system relapse, and 1 patient (1.5%) with testicular and BM relapse. The remaining 57 patients were in continuous CR.

**Fig. 3:**
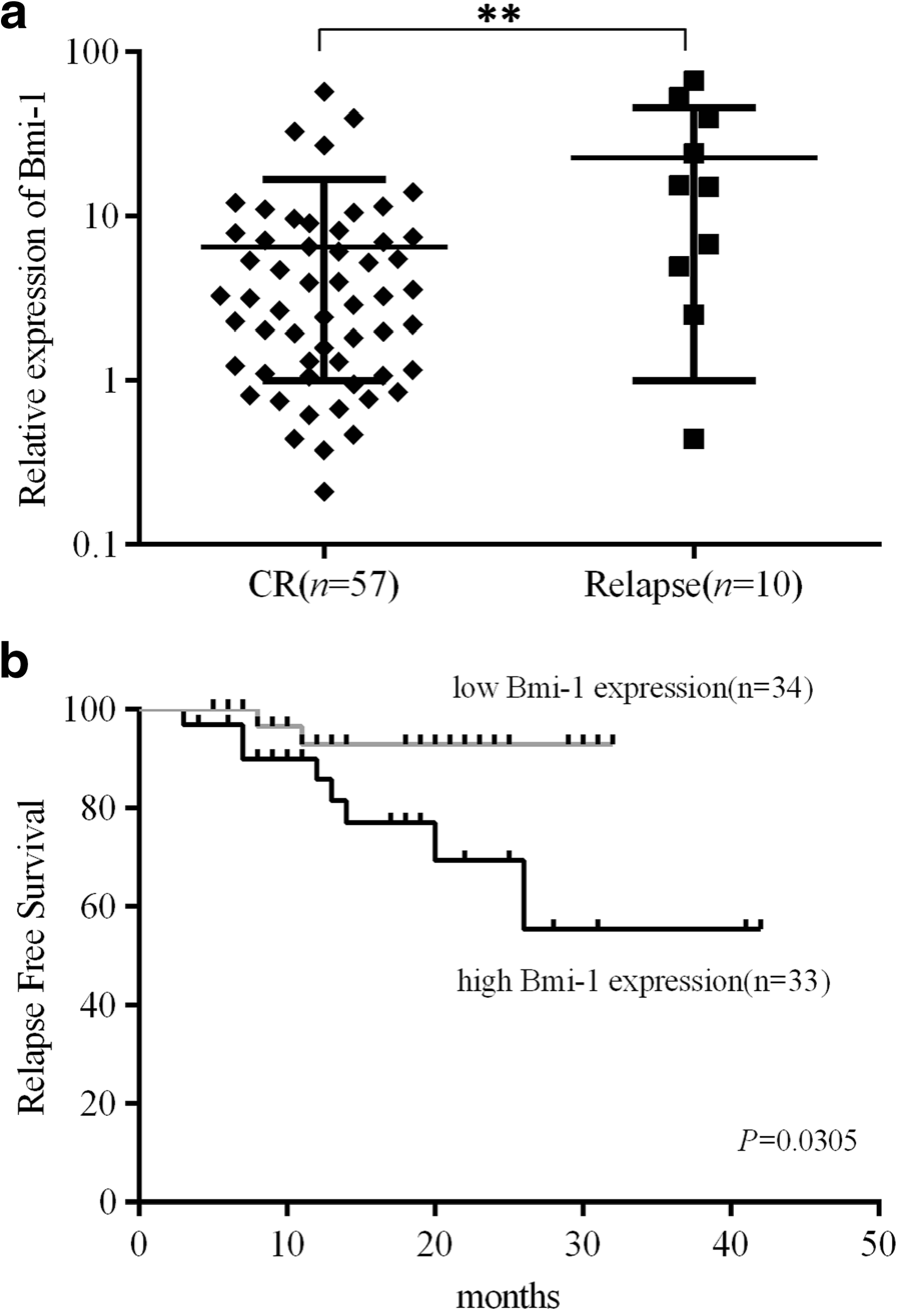
Bmi-1 expression was associated with ALL patient prognosis. **a** A total of 67 primary ALL patient samples were divided according to clinical outcomes. Patients who relapsed had significantly higher Bmi-1 expression levels than those who achieved CR after therapy. **b** The 3-year relapse-free survival (RFS) curves from a panel of 57 ALL patients. The cases were dichotomized based on the median expression of Bmi-1. Statistical differences between the curves were calculated by using the log-rank test, and the two-sided *P* value is indicated below the graph. **P < 0.01

After conducting a follow-up with these patients, we found that the Bmi-1 expression in the patients who relapsed (*n* = 10) was nearly 5-fold higher at the time of diagnosis than that of the remaining 57 patients with continuous CR (*P* < 0.01, Fig. 3a). The results indicated that Bmi-1 expression was related to leukemia relapse; thus, we hypothesized that Bmi-1 could act as a biomarker for predicting leukemia relapse. To confirm it, we detected the expression level of Bmi-1 in 57 newly diagnosed pediatric ALL patients using the same methods described above and found that the levels of Bmi-1 at the time of diagnosis were correlated to RFS. When the full set of 57 samples were divided into different expression groups, we observed that patients with Bmi-1 high-expressed at the time diagnosis had a lower RFS rate (75.8%) than patients with low expression of Bmi-1, who had an RFS rate of 94.1% (Fig. 3b).

### Elevated expression of Bmi-1 in pediatric ALL is associated with the expression of Sall4a

**Fig. 4:**
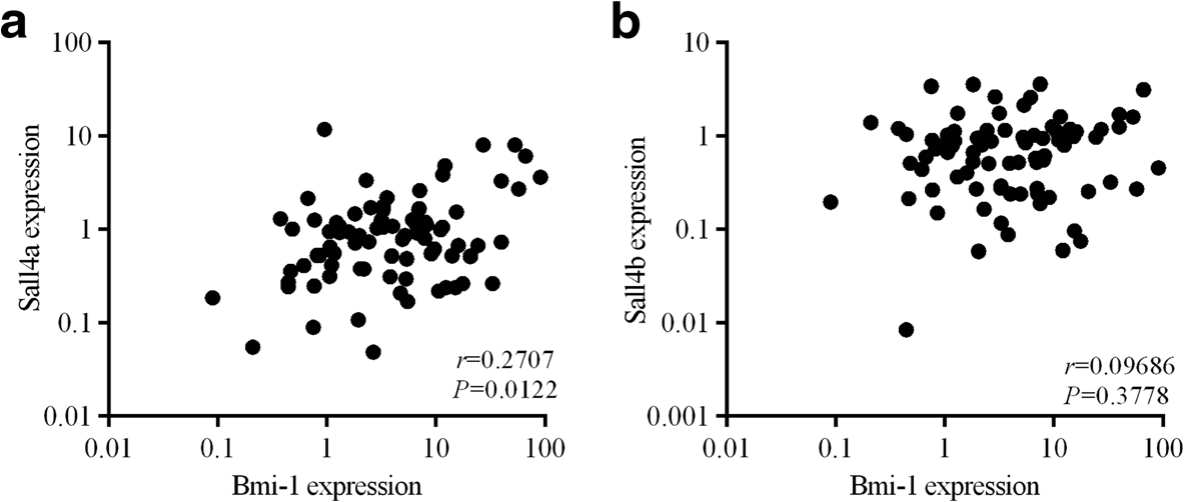
Bmi-1 expression was positively correlated with Sall4a expression in ALL patient samples. **a** A statistically significant positive correlation between the mRNA levels of Bmi-1 and Sall4a was observed in pediatric ALL specimens (r = 0.2707, *P* = 0.0122). **b** No statistically significant correlation between the mRNA levels of Bmi-1 and Sall4b was observed in pediatric ALL specimens (r = 0.09686, *P* = 0.3778)

It was previously reported that there was a significant relationship between the expression of Sall4 and Bmi-1 in AML samples. Based on this evidence, we measured the mRNA levels of Sall4 in pediatric ALL specimens and normal control tissues with the goal of identifying the possible mechanism that causes overexpression of Bmi-1 in ALL. We observed a significantly positive correlation between Bmi-1 expression and Sall4a (Fig. 4a, 2-tailed Spearman’s correlation, r = 0.2707; *P* = 0.0122); however, there was no statistical correlation between the Sall4b and Bmi-1 expression levels in the ALL samples examined (Fig. 4b, 2-tailed Spearman’s correlation, r = 0.09686; *P* = 0.3778). Furthermore, we found that there was no significant difference regarding the expression of Sall4a and Sall4b between pediatric ALL and normal control samples. In addition, there was also no difference between primary and CR groups (Additional file 7: Figure S2).

## Discussion

New biomarkers could be helpful in predicting treatment outcomes earlier and more precisely, which is of great interest to physicians and researchers in the field. This report describes for the first time that the proto-oncogene Bmi-1 is aberrantly expressed in the majority of primary ALL patients, and this expression is sharply decreased in CR patients after therapy. It has been shown that patients with elevated Bmi-1 expression at the time of diagnosis possessed a significantly higher likelihood of a poor response to prednisone and a higher clinical risk classification. Furthermore, we found that ectopic expression of Bmi-1 was closely associated with a poor prognosis for ALL patient survival, as patients with increased Bmi-1 expression had a significantly lower OS. Thus, this study not only extends our knowledge about the upregulation of this PcG protein but also verifies that Bmi-1 is an important and promising candidate tumor biomarker to predict the prognosis of pediatric patients with ALL.

There have been many studies that investigated the prognostic value of Bmi-1 expression in other types of tumors. Consistent with our results, research on ovarian cancer [35], breast cancer [36, 37], clear cell renal cancer [38], laryngeal carcinoma [39], cervical cancer [40], and esophageal adenocarcinoma [41] have reported an association between high Bmi-1 expression and an unfavorable prognosis. It has also been reported that high expression of Bmi-1 in AML cells is associated with an unfavorable prognosis [42]. In brief, Bmi-1 is at an important lynchpin in more than ten different types of cancer, and a wide spectrum of malignancies implicate Bmi-1 as a suitable candidate for predicting outcomes.

However, Teruyuki et al. [43] reported that Bmi-1 gene expression was lower in pediatric ALL and that there were no significant correlations between the Bmi-1 gene expression level in leukemic cells and clinical characteristics such as patient prognosis. These results were inconsistent with those of our study, which may be due to the different leukemia subtype and the limited number of samples. In Teruyuki’s study, the bone marrow-derived cells were obtained from 15 patients with pediatric precursor B-ALL that were sorted into different subsets by FACS, and CD19+ cells were treated as normal B cells for the analysis. In our study, we used mononuclear cells from the bone marrow instead of sorted normal B cells.

It has been well established that Bmi-1 is an essential regulator of cellular senescence [16, 44] and that overexpression of Bmi-1 could prevent the development of senescence in proliferating cells by directly repressing the expression of p16^Ink4a^ and p19^Arf^ [45]. Glucocorticoids then play a major role in apoptosis of hematopoietic cells including lymphocytes [46], exposure to the glucocorticoid dexamethasone results in changes for the expression of genes associated with cellular senescence, for example, upregulating cell cycle-related genes p16 and p21 [47]. Therefore, it is conceivable that Bmi-1 expression in ALL might counteract the effects of glucocorticoids on the cellular senescence pathway. Consequently, this could also explain why ALL patients with high Bmi-1 expression exhibited a poor response to prednisone.

Furthermore, our results also demonstrated that Sall4a and Sall4b, the two Sall4 isoforms, were constitutively expressed in pediatric ALL patients as well as in normal control subjects, although there was no statistically significant difference in these values between pediatric ALL samples and normal control samples. Similar to Bmi-1, Sall4 expression has been reported in numerous hematological malignancies, including myelodysplastic syndromes [48], AML [49, 50], chronic myelogenous leukemia [51] and precursor B cell lymphoblastic lymphoma [52, 53]. In addition, we found that the Bmi-1 gene expression levels showed a significantly positive correlation with Sall4a but not Sall4b. This result further verified the conclusion that a relationship between the Bmi-1 and Sall4 expression level in hematological malignancies, which was coincide with previously reports in AML samples [30, 31]. In addition, these findings indicate that Sall4a and Sall4b may have different functions in pediatric ALL. However, one would expect that Sall4a expression would be higher in primary ALL cells, but this was not the case in our study. We speculate that this discrepancy could be due to the weak correlation between Bmi-1 and Sall4a expression (r = 0.2707) and additional factors involved in the complex regulation of Bmi-1 expression. In addition, Sall4a expression was slightly higher in primary ALL cells, but this increase was not significant. Sall4a is upstream of Bmi-1, and little difference was observed between individuals because of the amplification of the downstream signaling cascade. To better clarify this effect, more experiments with a larger cohort are needed. However, the precise molecular mechanism of Bmi-1 in pediatric ALL still remains unclear and requires further elucidation.

## Conclusion

In summary, we provided evidence that Bmi-1 was significantly upregulated in pediatric ALL and that Bmi-1 overexpression was associated with a poor response to prednisone and a higher clinical risk. In addition, a significantly poorer outcome was observed in patients in the high Bmi-1 expression group. These findings suggest that Bmi-1 is an effective biomarker for predicting the prognosis of patients with pediatric ALL, and future studies should explore whether Bmi-1 could be a potential therapeutic target as well.

## Additional files

Additional file 1: Table S1. Detailed demographic characteristics of pediatric patients with ALL (*n* = 85). (XLSX 15 kb)
Additional file 2: Table S2. The demographic characteristics of healthy donors (*n* = 18). (DOCX 16 kb)
Additional file 3: Table S3. Sequences of the PCR primers and TaqMan probes. (DOCX 16 kb)
Additional file 4: Figure S1. The expression levels of Bmi-1 in pediatric ALL patients who achieved CR versus normal control subjects. The fold changes of data were presented with respect to the levels in the bone marrow from healthy donors (*n* = 18). ***P* < 0.01; CR, complete remission. (TIF 3 MB)
Additional file 5: Table S4. Details of treatment regimens for patients with pediatric ALL. (DOCX 20 kb)
Additional file 6: Table S5. Characteristics of the analyzed pediatric ALL subgroup. (DOCX 16 kb)
Additional file 7: Figure S2. The expression levels of Sall4 in pediatric ALL clinical specimens. (A) The average expression levels of Sall4a in pediatric ALL patients (*n* = 85) versus that in normal control subjects (*n* = 18), *P* = 0.0783. (B) The average expression level of Sall4b in pediatric ALL patients (*n* = 85) versus that in normal control subjects (*n* = 18), *P* = 0.2935. (C) The average expression levels of Sall4a before and after therapy (*n* = 19) in the paired samples from pediatric ALL patients, *P* = 0.3247. (D) The average expression levels of Sall4b before and after therapy (*n* = 19) in the paired samples from pediatric ALL patients, *P* = 0.8984. (JPG 1 MB)

## Abbreviations

ALL: Acute lymphoblastic leukemia
AML: Acute myeloid leukemia
Bmi-1: B cell-specific Moloney murine leukemia virus insertion site 1
CR: Complete remission
HR: High-risk
HSC: Hematopoietic stem cell
IR: Intermediate risk
LR: Low-risk
PcG: Polycomb-group
qRT-PCR: Quantitative reverse transcription polymerase chain reaction
RFS: Relapse-free survival
WBC: White blood cell count
