# Supplementary material for: Upregulation of the proto-oncogene Bmi-1 predicts a poor prognosis in pediatric acute lymphoblastic leukemia
Source: BMC Cancer. 2017 Jan 25;17:76. doi: 10.1186/s12885-017-3049-3 (PMC5264321; doi:10.1186/s12885-017-3049-3)
Supplement: Additional file 2: Table S2. — The demographic characteristics of healthy donors (n = 18). (DOCX 16 kb) [file 12885_2017_3049_MOESM2_ESM.docx]

**Additional file 2: Table S2 The characteristics of healthy donors (n=18)**

| **Characteristics** | **Median(range)** | **No.(%)** |
| --- | --- | --- |
| **Age, year** | **8(6-12)** |  |
| **Gender** |  |  |
| **Male** |  | **10(55.6)** |
| **Female** |  | **8(44.4)** |
| **WBC count(×10^9^/L)** | **6.8(5.9-10.4)** |  |
