# Supplementary material for: Upregulation of the proto-oncogene Bmi-1 predicts a poor prognosis in pediatric acute lymphoblastic leukemia
Source: BMC Cancer. 2017 Jan 25;17:76. doi: 10.1186/s12885-017-3049-3 (PMC5264321; doi:10.1186/s12885-017-3049-3)
Supplement: Additional file 3: Table S3. — Sequences of the PCR primers and TaqMan probes. (DOCX 16 kb) [file 12885_2017_3049_MOESM3_ESM.docx]

Additional file 3: Table S3 Sequences of the PCR primers and Taqman probes

| Gene | Sequence |
| --- | --- |
| Bmi-1 | F： CTGGTTGCCCATTGACAGC |
|  | R： CAGAAAATGAATGCGAGCCA |
|  | Probe: CAGCTCGCTTCAAGATGGCCGC |
| Sall 4a | F： CCCTTCGTGTGCTCTGTCTG |
|  | R： CGCCACTTTGTCCTGGAACT |
|  | Probe: TCATCGCTTCACCACCAAGGGC |
| Sall 4b | F： GCACAAGTGTCGGAGCAGTC |
|  | R： GTGGCTGGGCTGCTAACAA |
|  | Probe: CCTTCCACGTTTATCCGAGCCC |
| GAPDH | F： CTCCTCCTGTTCGACAGTCAG |
|  | R： CCCAATACGACCAAATCCGTT |
|  | Probe: CATCACTGCCACCCAGAAGACTGTG |

F: Forward Prime；R, Reverse Prime.
