# Supplementary figures and images for: Upregulation of the proto-oncogene Bmi-1 predicts a poor prognosis in pediatric acute lymphoblastic leukemia
Source: BMC Cancer. 2017 Jan 25;17:76. doi: 10.1186/s12885-017-3049-3 (PMC5264321; doi:10.1186/s12885-017-3049-3)

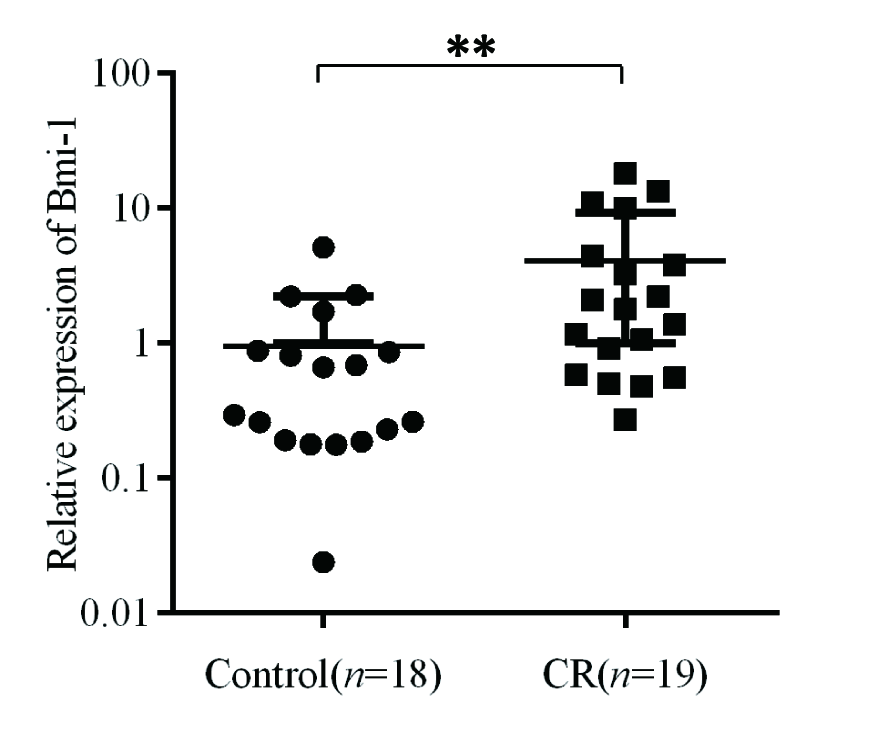

Supplement: Additional file 4: Figure S1. — The expression levels of Bmi-1 in pediatric ALL patients who achieved CR versus normal control subjects. The fold changes of data were presented with respect to the levels in the bone marrow from healthy donors (n = 18). **P < 0.01; CR, complete remission. (TIF 3 MB) [file 12885_2017_3049_MOESM4_ESM.tif]

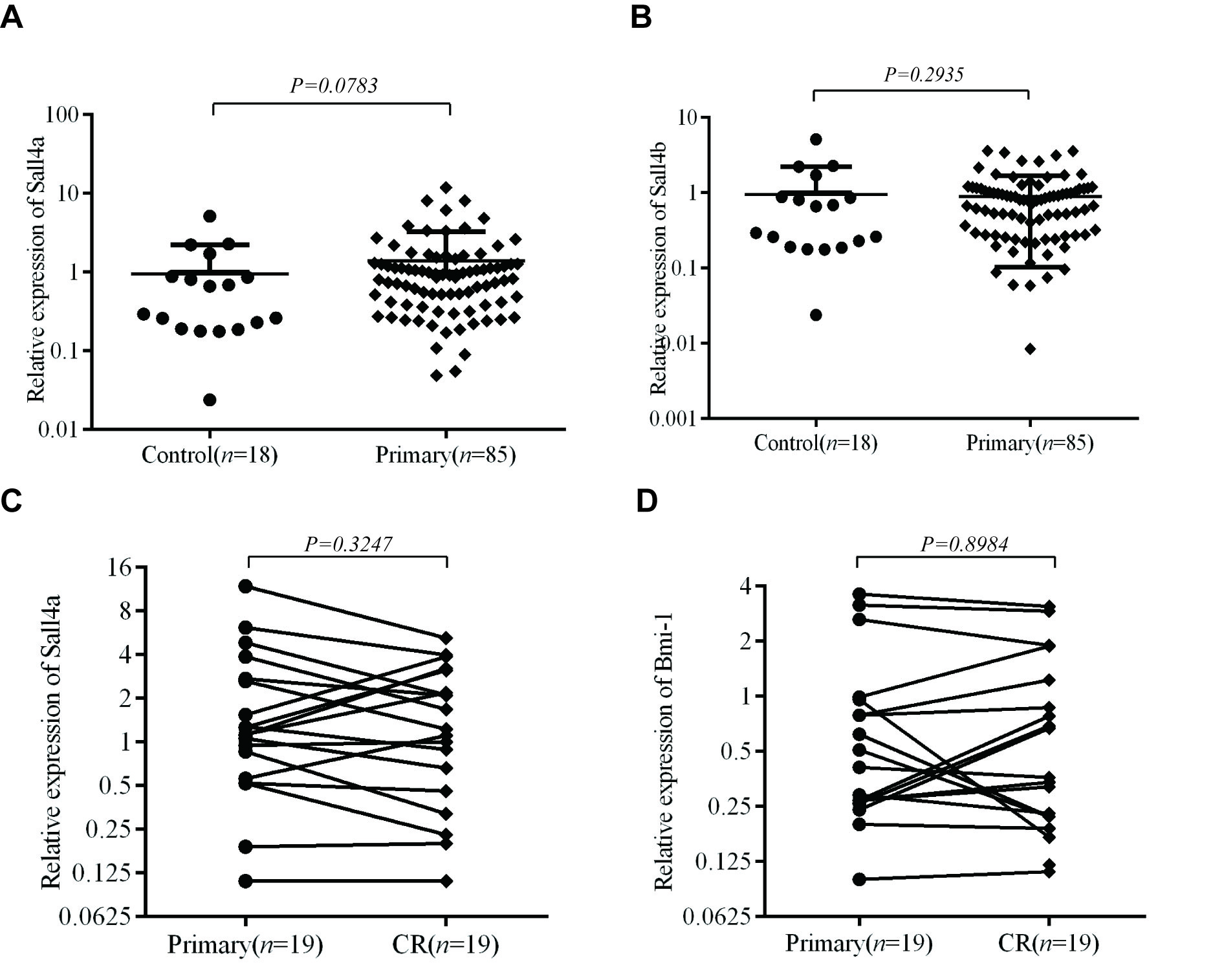

Supplement: Additional file 7: Figure S2. — The expression levels of Sall4 in pediatric ALL clinical specimens. (A) The average expression levels of Sall4a in pediatric ALL patients (n = 85) versus that in normal control subjects (n = 18), P = 0.0783. (B) The average expression level of Sall4b in pediatric ALL patients (n = 85) versus that in normal control subjects (n = 18), P = 0.2935. (C) The average expression levels of Sall4a before and after therapy (n = 19) in the paired samples from pediatric ALL patients, P = 0.3247. (D) The average expression levels of Sall4b before and after therapy (n = 19) in the paired samples from pediatric ALL patients, P = 0.8984. (JPG 1 MB) [file 12885_2017_3049_MOESM7_ESM.jpg]
