# Supplementary material for: Upregulation of the proto-oncogene Bmi-1 predicts a poor prognosis in pediatric acute lymphoblastic leukemia
Source: BMC Cancer. 2017 Jan 25;17:76. doi: 10.1186/s12885-017-3049-3 (PMC5264321; doi:10.1186/s12885-017-3049-3)
Supplement: Additional file 5: Table S4. — Details of treatment regimens for patients with pediatric ALL. (DOCX 20 kb) [file 12885_2017_3049_MOESM5_ESM.docx]

Additional file 5: Table S4 The details of treatment regimens for pediatric ALL

|  | **ALL IC-BFM 2002** | |  | **VHR-ALL GZCLC** |
| --- | --- | --- | --- | --- |
|  | **Low-risk ALL protocol** | **Intermediate risk ALL protocol** |  | **High-risk ALL protocol** |
| **Induction 1** | | | | |
| Prednisone 60mg/m^2^ PO d1-7  Dexamethasone 6mg/m^2^ PO d8-29  VCR 1.5mg/m^2^ IV d8,15,22,29  DNR 30mg/m^2^ IV d8,15  L-Asp 5000U/m^2^ IV  d12,15,18,21,24,27,30,33 | | Prednisone 60mg/m^2^ PO d1-7  Dexamethasone 6mg/m^2^ PO d8-29  VCR 1.5mg/m^2^ IV d8,15,22,29  DNR 30mg/m^2^ IV d8,15  L-Asp5000U/m^2^ IV  d12,15,18,21,24,27,30,33 | | Prednisone 60mg/m^2^ PO d1-7  Dexamethasone 6mg/m^2^ PO d8-29  VCR 1.5mg/m^2^ IV d8,15,22,29  DNR 30mg/m^2^ IV d8,15  L-Asp5000U/m^2^ IV  d12,15,18,21,24,27,30,33 |
| **Induction 2** | | | | |
| CTX 1g/m^2^ IV d1,28  Ara-C75mg/m^2^  SC d3-6,10-13,7-20,24-47  6MP 25mg/m^2^ PO d1-56 | | CTX 1g/m^2^ IV d1,28  Ara-C 75mg/m^2^ SC d3-6,10-13,7-20,24-47  6MP 25mg/m^2^ PO d1-56 | | |
| **Consolidation 1** | | | | |
| # MTX 2g/m^2^ or 5g/m^2^ IV d8,22,36,49  6MP 25mg/m^2^ PO d1-56 | | # MTX 2g/m^2^ or 5g/m^2^ IV d8,22,36,49  6MP 25mg/m^2^ PO d1-56 | | Ara-C 1.5g/m^2^ IV q12h d1-3  MA 10mg/m^2^ IV d2-3  L-ASP 10000U/m^2^ IV d5,8,11,14  Repeat one course when hemogram recovers |
| **Consolidation 2** | | | | |
|  | | | | MTX 5g/m^2^ IV d2,12,23  6MP 75mg/m^2^ PO d1-7,11-17,22-28 |
| **Reinduction 1** | | | | |
| Dexamethasone PO 10mg/m^2^ d1-21  VCR 1.5mg/m^2^ IV d8,15,22,29  ADM 30mg/m^2^ IV d8,15,22,29  L-Asp 10000U/m^2^ d8,11,14,17 | | Dexamethasone PO 10mg/m^2^ d1-21  VCR 1.5mg/m^2^ IV d8,15,22,29  ADM 30mg/m^2^ IV d8,15,22,29  L-Asp 10000U/m^2^ d8,11,14,17 | | CTX 1g/m^2^ IV d1  Ara-C 1g/m^2^ IV q12h d2-4  6MP 50mg/m^2^ PO d1,4,7 |
| **Reinduction 2** | | | | |
| CTX 1g/m^2^ IV d1  Ara-C 75mg/m^2^ SC d3-6,10-13  6MP 60mg/m^2^ PO d1-15 | | CTX 1g/m^2^ IV d1  Ara-C 75mg/m^2^ SC d3-6,10-13  6MP 60mg/m^2^ PO d1-15 | | Eptoposide 300mg/m^2^ IV d1,4,7  Ara-C300mg/m^2^ IV d1,4,7 |
| **&Maintenance** | | | | |
| MTX 20mg/m^2^ PO weekly  6MP 50mg/m^2^ PO daily | | MTX 20mg/m^2^ PO weekly  6MP 50mg/m^2^ PO daily | | MTX 20mg/m^2^ PO weekly  6MP 50mg/m^2^ PO daily |
| **Intensiflcation** | | | | |
|  | | | | Dexamethasone 15mg/m^2^ PO d1-7  CTX 1g/m^2^ IV d1  VCR 1.5mg/m^2^ IV d1  Ara-C 75mg/m^2^ SC q12h d1-7  Administered at the end of 3^nd^ month from  the beginning of maintenance |
