# Supplementary material for: Upregulation of the proto-oncogene Bmi-1 predicts a poor prognosis in pediatric acute lymphoblastic leukemia
Source: BMC Cancer. 2017 Jan 25;17:76. doi: 10.1186/s12885-017-3049-3 (PMC5264321; doi:10.1186/s12885-017-3049-3)
Supplement: Additional file 6: Table S5. — Characteristics of the analyzed pediatric ALL subgroup. (DOCX 16 kb) [file 12885_2017_3049_MOESM6_ESM.docx]

| **Parameters** | **Treatment group** | **Give up the treatment group** | ***P* Value** |
| --- | --- | --- | --- |
|  | ***n*(%), *N*=67** | ***n*(%), *N*=18** |  |
| **Age at diagnosis, y** |  |  | 056 |
| **＜6** | 35 | 8 |  |
| **≥6** | 32 | 10 |  |
| **Gender** |  |  | 0.64* |
| **Male** | 46 | 14 |  |
| **Female** | 21 | 4 |  |
| **WBC count(×10^9^/L)** |  |  | 0.50 |
| **＜50** | 43 | 10 |  |
| **≥50** | 24 | 8 |  |
| **FAB classification** |  |  | 0.88* |
| **L1** | 26 | 8 |  |
| **L2** | 38 | 9 |  |
| **L3** | 3 | 1 |  |
| **Immunophenotype** |  |  | 0.49* |
| **T** | 9 | 2 |  |
| **B** | 53 | 13 |  |
| **Unknown** | 5 | 3 |  |
| **BCR/ABL** |  |  | 0.27* |
| **+** | 5 | 3 |  |
| **-** | 53 | 11 |  |
| **Unknown** | 9 | 4 |  |

**Additional file 6: Table S5 Characteristics of the analyzed pediatrics ALL subgroup**

*Two-sided Fisher’s exact test.
